# Early Studies of Binocular and Binaural Directions

**DOI:** 10.3390/vision2010013

**Published:** 2018-03-01

**Authors:** Nicholas J. Wade

**Affiliations:** Department of Psychology, University of Dundee, Dundee DD1 4HN, UK; n.j.wade@dundee.ac.uk

**Keywords:** vision, audition, visual direction, auditory localization, stereoscope, pseudoscope, stethophone, pseudophone, Wells, Venturi

## Abstract

Understanding how the eyes work together to determine the direction of objects provided the impetus for examining integration of signals from the ears to locate sounds. However, the advantages of having two eyes were recorded long before those for two ears were appreciated. In part, this reflects the marked differences in how we can compare perception with one or two organs. It is easier to close one eye and examine monocular vision than to “close” one ear and study monaural hearing. Moreover, we can move our eyes either in the same or in opposite directions, but humans have no equivalent means of moving the ears in unison. Studies of binocular single vision can be traced back over two thousand years and they were implicitly concerned with visual directions from each eye. The location of any point in visual or auditory space can be described by specifying its direction and distance, from the vantage point of an observer. From the late 18th century experiments indicated that binocular direction involved an eye movement component and experimental studies of binaural direction commenced slightly later. However, these early binocular and binaural experiments were not incorporated into theoretical accounts until almost a century later. The early history of research on visual direction with two eyes is contrasted to that on auditory direction with two ears.

## 1. Introduction

History has looked more favorably on vision than on audition, if favor is measured by the pages devoted to each in texts on the senses (see Table 1). Contrasts between seeing and hearing can be considered in terms of the amount of space each occupies in books on the senses. For example, in surveys of Greek theories more than twice the space is given to vision than hearing [1,2] and the bias towards vision was even greater for Galen [3]. Hearing did not fare any better in the medieval period [4,5] and at the dawning of the scientific revolution Aquapendente’s [6] book on vision and audition gave precedence to the former. The situation was little changed over the next centuries. In his book on the nervous system and the senses, Bell [7] devoted almost twice as many pages to vision as to hearing. Even though research on hearing increased enormously in the 19th century this was not reflected in the books surveying this period [8,9]. However, Luciani’s survey was among the first to include a section on binaural hearing. A wider range of auditory phenomena was given by Boring [10] in his book on the senses but the disparity was maintained; it did contain a section on auditory localization. By the late 20th century the bias in favor of vision had increased [11,12]. More recently, the ratio of pages on vision to hearing was about 3:1 in Kandel, et al. [13] and in Goldstein [14].

The exception to this pattern was a book by Pierce [15] which was restricted to visual and auditory space perception: 200 pages were devoted to auditory localization in contrast to 149 for vision. Pierce conducted many studies on auditory localization which are fully described in his book but the section on vision was confined to a series of accounts of visual spatial illusions and very little attention was given to visual direction. An historical section is given for auditory space but not for visual space. Unfortunately, the history of research on binaural hearing is said to start with Weber [16] whereas experiments were conceived of and conducted half a century earlier [17,18].

Why has vision been so favored historically? It could relate to the knowledge about the stimulus for hearing (sound) in contrast to the ignorance about the nature of light. One consequence of this is that the study of vision was observational (and psychological) whereas that for audition was essentially physical. Additional factors relate to the sense organs themselves: the eyes can move, often in opposite directions, whereas the ears (in humans) require movements of the head to change their direction. Moreover, theories of vision have incorporated concepts concerned with spatial images for which there was no equivalent in audition.

**Figure 1:**
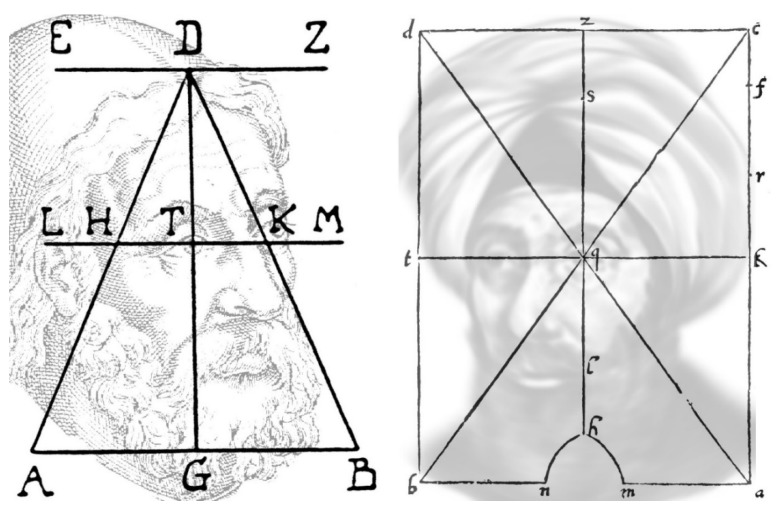
*The boards of Ptolemy and Alhazen* by Nicholas Wade. Portraits of Ptolemy (**left**) and Alhazen (**right**) are combined with diagrams of the boards they constructed to examine binocular direction. The eyes were placed at positions denoted by A and B with fixation on the points D and q, respectively.

This distinction is even more pronounced in the domain of spatial localization. Determining where an object is located with respect to an observer is essential for survival and vision has been accorded precedence over hearing to achieve this. Euclid’s geometrical theory of vision was predicated on visual direction and it is an essential element in Kepler’s projection theory [19]. Experimental investigations of binocular direction were undertaken by both Ptolemy in the -2nd century [20,21] and Ibn al-Haytham or Alhazen in the 11th [22,23] using boards upon which stimuli could be placed (Figure 1). Ptolemy concluded that an object on the common axis nearer than the fixation point would appear double, and that objects on the visual axes are seen in three locations: fused on the common axis and laterally separated by twice the distance between the visual axes. These principles of binocular visual direction were redescribed by Ibn al-Haytham and rediscovered by Wells [24] and by Hering [25].

Over this large timescale, very little was written about binaural hearing, in contrast to the wealth of binocular phenomena that was discussed and investigated experimentally. Things were to change fundamentally in the 19th century both in terms of the instruments that can differentially stimulate two eyes or two ears and the way the new phenomena were interpreted [17,18,26,27]. The initial concern was not with binaural direction but whether binaural beats could be perceived when different sounds (like ticking watches) were presented to each ear. The comparison was between binocular and binaural rivalry.

**Figure 2:**
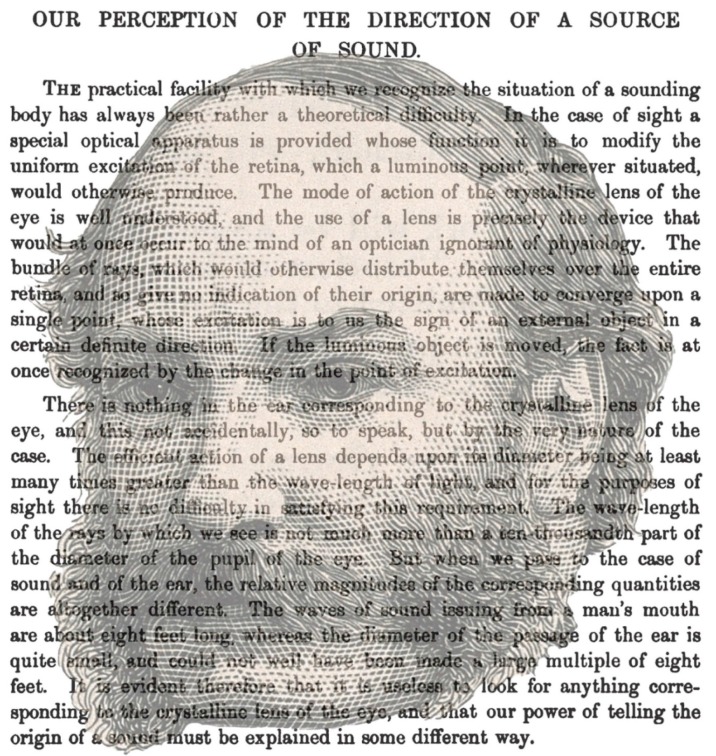
*Binaural theorist* by Nicholas Wade. A portrait of Lord Rayleigh (John William Strutt) combined with text from his article on auditory localization [40].

The divergent histories of binocular vision and binaural hearing are also reflected in the terminologies associated with them and the times at which were introduced. This relates to the instruments that were devised to stimulate the paired organs. Binocular instruments have been made since the 16th century and the study of binocular vision was revolutionized by the invention of the stereoscope by Wheatstone [28,29]; either paired mirrors or prisms enabled slightly different stimuli to be presented to each eye. The auditory equivalent of the stereoscope was invented by Alison [30] and it was called a stethophone. It consisted of independent ear tubes so that different sounds could be listened to. He was not stimulated to study binaural hearing based on Wheatstone’s stereoscope, but because of his experiments in audition [18]. Alison’s experiments mostly involved two watches and he formulated two laws: “1st, that sounds of the same character are restricted to that ear into which they are conveyed in greater intensity, and 2nd, that sounds differing in character may be heard at the same time in the two ears respectively, even if they be made to reach the ears in different degrees of intensity” ([30], p. 205). Alison [31] later referred to the stethophone as the bin-aural stethoscope. Experiments on binaural direction commenced in earnest in the 1870s [26,27]. In this decade, Rayleigh ([32,33]; Figure 2) reported his examinations of auditory localization, Thompson [34,35,36] investigated a range of binaural phenomena and invented the pseudophone, and Steinhauser [37,38,39] published a formal appreciation that hearing with two ears differed from that with one. Like most early contributors to binaural hearing, Steinhauser’s initial interests were in binocular vision. He wrote extensively on stereoscopic photography as well as publishing what was perhaps the first monograph on binaural hearing [37]; it was translated into English by Thompson and published two years later. Steinhauser stated: “The theory of Audition may be divided into two portions—that of Monaural Audition, or of hearing with one ear, and that of Binaural Audition, or of hearing with both ears. The former, already treated in every textbook of Physics, is concerned with the arrangement of the human ear, the function of its separate parts, and, lastly, how the ear is instrumental in the faculty of hearing. The second branch of the subject, which has never, to my knowledge, been yet developed, must discuss the general question of hearing, with respect in particular to the circumstance that it is performed with two ears. It is concerned, further, in deciding what part binaural hearing plays in the various phenomena of hearing in general, and the various advantages thereby gained” ([38], pp. 181–182).

## 2. Binocular Direction

**Figure 3:**
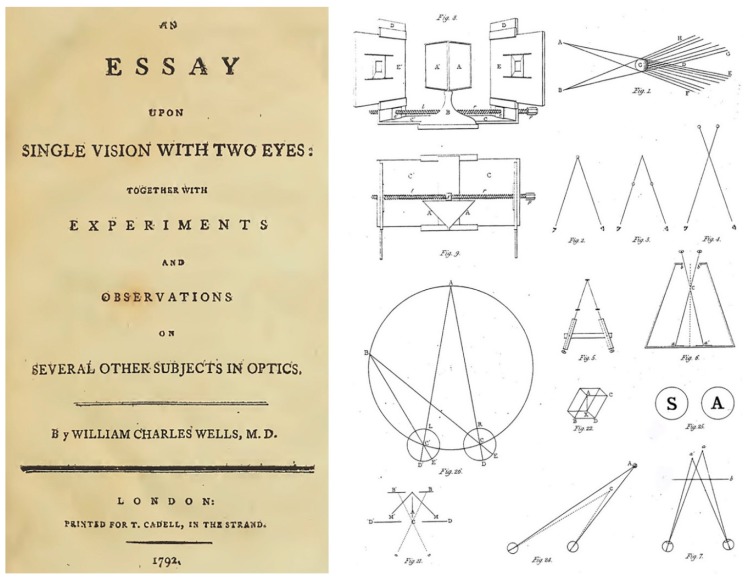
(**Left**) the title page from Wells [24] and (**right**) Plate 1 from Wheatstone [28].

Binocular vision can be considered as providing information for two vital aspects of adaptation to the environment—the direction in which objects appear to us and the distances between them. These two dimensions have not been accorded equivalent prominence in the history of vision research. Since the invention of the stereoscope attention has focused principally on perceived depth or distance. Prior to that, Wells [24] had conducted experiments on binocular visual direction; his book was the first dedicated solely to binocular vision (Figure 3). The revolution in binocular vision that occurred after Wells (in the early 1830s) was occasioned by Wheatstone’s invention and application of the stereoscope to demonstrate depth from retinal disparities. The first plate from his paper is shown in Figure 3; it not only illustrates his mirror stereoscope but also other methods of combining different images in each eye. The stereoscope, perhaps more than any other instrument, ushered in the era of experimentation to vision. It fulfilled Wells’ desire to examine binocular vision by observation and experiment, but it was concerned with visual depth rather than visual direction.

Experimental and theoretical interest in visual direction has increased in the last three decades and a greater appreciation of the pioneering research of Wells is evident [19,41,42,43,44,45]. Wells commenced his analysis of visual direction with a critical assessment of optical theories of Aguilonius [46], who introduced one of the seminal ideas in the experimental study of binocular single vision—the horopter. Allied to this was the concept of binocular single vision mediated by a single location between the two eyes. In his customary style, Wells introduced the concept of the horopter only to point out difficulties in the way it was conceived of by Aguilonius. Despite this Wells does give due regard to Aguilonius for integrating binocular single and double vision.

Visible direction (as it was referred to in the 18th century) is a central concept in experimental and philosophical analyses of space perception. It is the aspect that is readily available and one that can be relatively easily assessed. Ptolemy applied it to the analysis of binocular vision, as did Galen, and when the principles of image formation in the eye were understood (in the early 17th century), it could be linked to optics. However, the eyes are very mobile within their orbits and so the involvement of eye movements in the perception of visual direction posed obvious problems. In this regard Wells was stimulated by the writing of Porterfield [47,48] on the integration of eye movements and vision. Eye movements were considered important because of the function they served, and Porterfield, in his two long essays on the motions of the eyes, made many digressions into areas of perception when treating them. Although he was purporting to examine eye movements in the essays, his principal aim was philosophical. For Porterfield both direction and distance were considered to be innate, whereas for Reid [49] it was the monocular (two-dimensional) visible direction that was inborn. This principle of visual direction was attacked by Wells on empirical grounds. First, it could not predict the diplopia experienced by individuals with strabismus after they have aligned an object in each eye separately. Secondly, it could not account for the apparent direction of afterimages following voluntary movements of the eyes. Wells refined Porterfield’s argument by separating situation (perceived location) into visual direction and visual distance and proposing that the former is innate whereas the latter is learned. Wells provided an alternative to the extreme views of philosophical nativists and empiricists.

**Figure 4:**
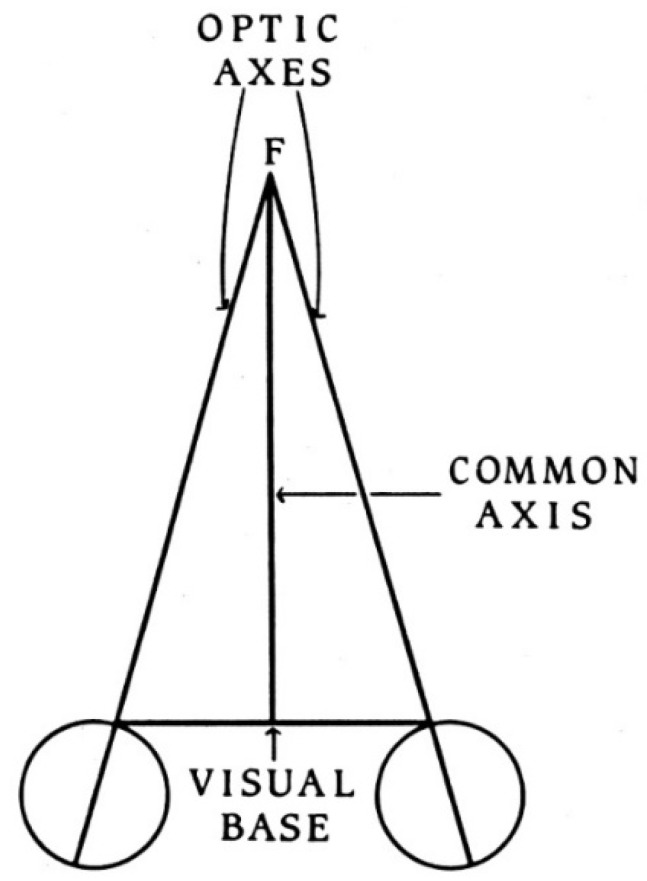
A diagram illustrating the terms used by Wells to describe the phenomena of visual direction (from [44]).

Having expressed dissatisfaction with the two classes of explanation previously given for binocular single vision, Wells proceeded to present his own analysis and a novel theory. In order to achieve this, he is assiduous in defining the terms he will employ. For example, the optic axis is determined by means of a visual alignment task—when a small, fixated target can be obscured by a closer small object then the optic axis is the line joining them and the eye. The visual base is essentially the inter-ocular separation, and Wells adopts Alhazen’s definition of the common axis. These were described by Wells in his text, but he did not provide any figures to illustrate them; they are shown diagrammatically in Figure 4.

There followed three propositions regarding consequences of viewing lines with one or two eyes in the optic or common axes. Thus, if an object is viewed through two small holes, one in front of each eye, the object will be perceived to lie in the common axis. This point was not novel and was not contested. However, unlike earlier commentators, Wells considered the perception of the holes as well as the object: the holes appear single and in the common axis, too. When the holes were moved further from the eyes they are still perceived as a single hole in the common axis.

This proposition was amplified in terms of an experiment with lines or threads drawn along the optic axes of one or both eyes. When a pin, to which a string is attached in the optic axis of one eye, is viewed by that eye the line appears in the common axis. When a distant point on a line in the common axis is fixated, the line appears in the direction of the closed eye. When two differently colored lines are presented in the optic axes of the eyes then viewing the pin results in the visibility of three lines; that in the common axis fluctuates between the two colors, whereas the other two are seen as directed temporally of each eye. The fluctuation of the colors of the line in the common axis reflects an instance of binocular color rivalry, and Wells provided a long footnote describing some of the earlier observations of this phenomenon. In the course of the footnote mention is made of presenting different sounds to each ear analogously to presenting different colors to each eye. This is one of the earliest references to what is now called dichotic listening, although it was many decades before this phenomenon was examined experimentally.

**Figure 5:**
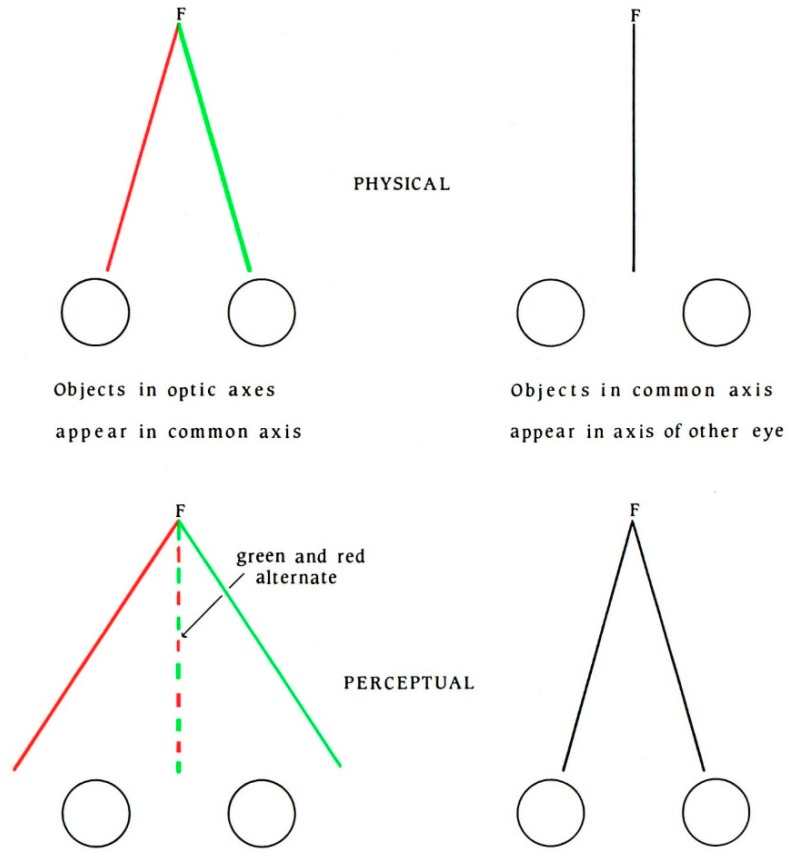
A diagrammatic representation of Wells’ propositions of visual direction when viewing strings of different colors.

Wells did not provide any diagrams to illustrate these conditions, which are shown schematically in Figure 5. Ono [42] constructed several figures to represent Wells’ three propositions of visual direction. Ono suggested that one of the possible reasons for the neglect of Wells’ theory was the absence of any illustrations in his *Essay*, which would have assisted a reader in understanding the conditions he described.

**Figure 6:**
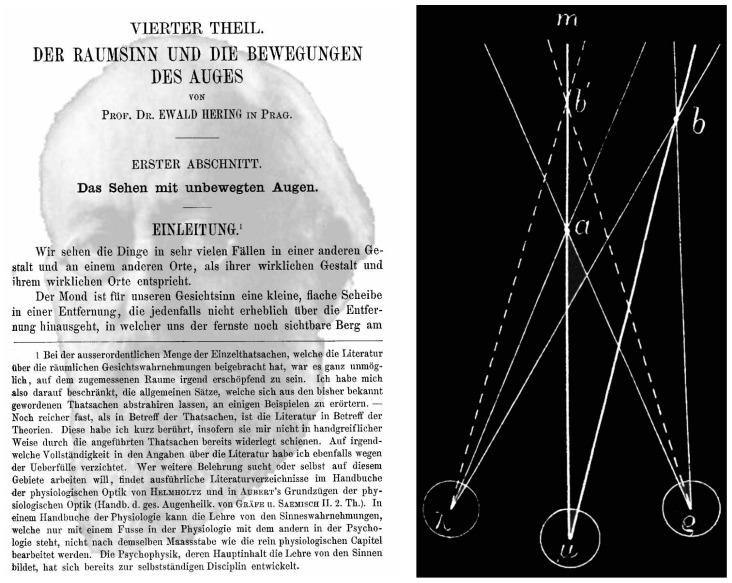
(**Left**) *Hering’s spatial sense* by Nicholas Wade. Ewald Hering is shown in text from his chapter on the spatial sense and eye movements [25]; (**Right**) Hering’s diagram from the same chapter describing viewing a point on a window with different objects aligned with it and each eye.

When Hering (Figure 6, left) turned to visual direction Wells had been forgotten. Hering essentially rediscovered the principles of visual direction described by Wells, although no reference was made to Wells’ earlier enquiries [42,50]. Ono [42] has illustrated the difference between the systems of Wells and Hering. The predictions are the same, but the concepts employed in reaching those identical predictions introduce some subtle distinctions. Hering made recourse to the classical concept of the cyclopean eye (without using that term). This was, of course, known to Wells, but he did not use it as it added little to the predictions he could make regarding visual direction. Unlike Wells, Hering provided plentiful illustrations to support his analyses of visual direction. One description that is frequently illustrated is that of viewing objects through a window: an object on the right is viewed by the left eye and a mark is made on the window, this mark is then aligned with an object on the left viewed by the right eye alone. When both eyes view the mark the two objects appear straight ahead as if aligned with the cyclopean eye. Hering’s illustration is shown in Figure 6 (right) but subsequent representations have included the tree and house as in Hering’s description [41].

The ignorance of earlier work in English attests to the ascendancy of German science in the second half of the 19th century. If a work was not written in German, or was not translated into that language, it would not have been considered of significance. Since Wells’ writing was ignored by most English-speaking scientists there is little wonder that it was given even less attention by others.

## 3. Binaural Direction

**Figure 7:**
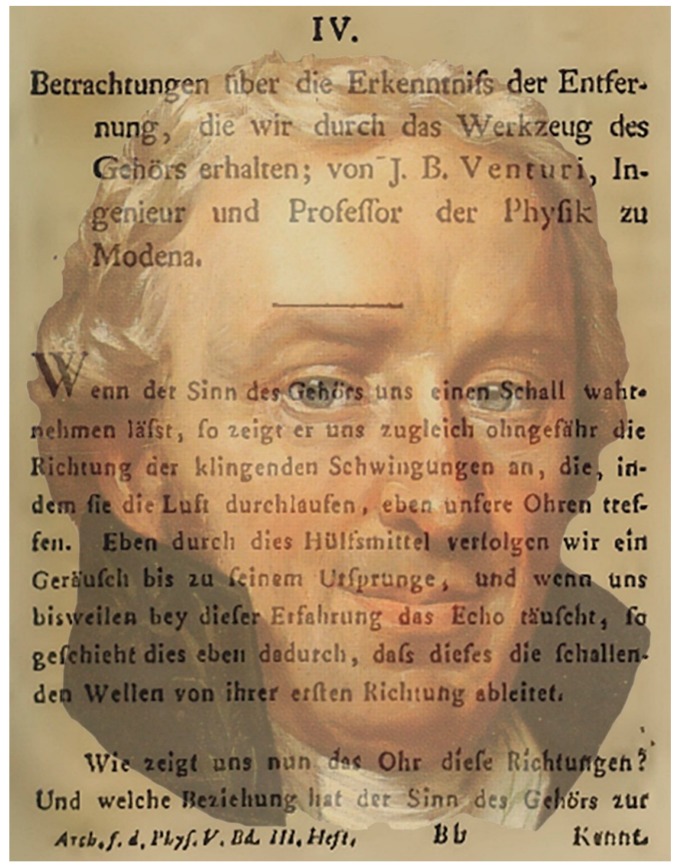
*Venturi’s vision* by Nicholas Wade. A portrait of Giovanni Battista Venturi in text from his article on binaural direction [52].

In the same decade that Wells presented his analysis of binocular direction Venturi ([51,52]; Figure 7) reported his experimental investigations of auditory localization, comparing listening with both ears or with one blocked by a finger. A blindfolded listener stood on a flat and unbounded surface and notes from a flute were played from various directions at a distance of 40–50 m. In his first study one ear was stopped by a finger. Sounds could be located when they were perpendicular to the open ear. This direction was called the auditory axis, following the concept of the visual axis. His second study was also with one ear stopped but the blindfolded listener turned until the sound was loudest. This occurred when the sound was in the auditory axis of the open ear. The third study was with both ears open and a stationary head. The listener was able to determine with reasonable accuracy the direction of a sound, but this could not be maintained when one ear was stopped with a finger. Partially blocking one ear changed the apparent direction of the sound. On the basis of this observation Venturi stated: “Therefore the inequality of the two impressions, which are perceived at the same time by both ears, determines the correct direction of the sound” ([52], p. 186). Venturi also established that a listener with both ears open could not distinguish between a sound directly in front of them or behind.

In his wide-ranging lectures on natural philosophy Young [53] mentioned Venturi’s experiments on hydraulics but not on hearing. Young was aware of the selective nature of hearing, and also of the phenomenon of auditory localization, but the means by which the latter was achieved was considered to be an enigma: “Thus, we can distinguish very accurately the voices of our friends, even when they whisper, and those modifications of the same voice which constitute the various vowels and semivowels, and which, with the initial and final noises denominated consonants, compose the words of language. We judge also, without an error of many degrees, of the exact direction in which the sound approaches us; but respecting the manner in which the ear is enabled to make this discrimination, we cannot reason upon any satisfactory grounds” ([53], p. 388). It took many decades before such grounds would be based on experimental evidence.

Almost 70 years after Venturi’s experiments, Rayleigh [32] performed a similar study, but in ignorance of its predecessor. Rather than move around a listener (because the footsteps could be detected), he placed assistants in several directions and they produced sounds when instructed: “The uniform result was that the direction of a human voice used in anything like a natural manner could be told with certainty from a single word, or even vowel, to within a few degrees” ([32], p. 32). Similar results were found with tuning forks, although sounds from directly ahead or behind were confused. Differences between the intensities of sounds at each ear were thought to be involved, but calculations of the differences led him to question whether they were large enough to account for the power of discrimination.

It was at the Plymouth meeting of the British Association for the Advancement of Science that Thompson [34] described his experiments on binaural beats: “Two tuning-forks tuned nearly in unison when sounded together give rise to interference “beats”. These “beats” are heard also if the forks be held one to each ear, or if their sounds be conveyed separately to the ears with pipes” (pp. 37–38). More detailed accounts were presented in three subsequent articles [36,54,55]. He was among the first to use the term “binaural audition”. Thompson [56] examined auditory localization in the context of visual localization. Both were analyzed in terms of direction and distance and he noted the differences between ears and eyes in terms of focusing, receptor layout, and motor control. The features involved in auditory localization were listed: “There are four physical characteristics of waves of sound by which one sound is discriminated from another, viz: (i) *Intensity*, or loudness, depending upon extent or energy of the vibratory motions; (ii) *Pitch*, or frequency, depending upon the rapidity of the vibratory motions; (iii) *Phase* of the vibratory motions, as to whether moving backward or forward or at any other state; (iv) *Quality*, or timbre, depending upon the degree of complexity of the vibratory motion. The third of these physical characteristics is one for which the single ear possesses no direct means of perception” ([56], p. 408, original italics). Thus, Thompson argued that phase differences alone were in the province of binaural hearing and so served the function of localizing the direction of sounds in space. Distance presented a more complex problem, and he considered that: “In the case of known sounds we doubtless judge chiefly of their distance by their relative loudness, the intensity decreasing inversely as the square of the distance” ([56], p. 415). Nonetheless, Thompson did entertain the possibility of “acoustic parallax” playing a role in its determination for sounds at short distances.

**Figure 8:**
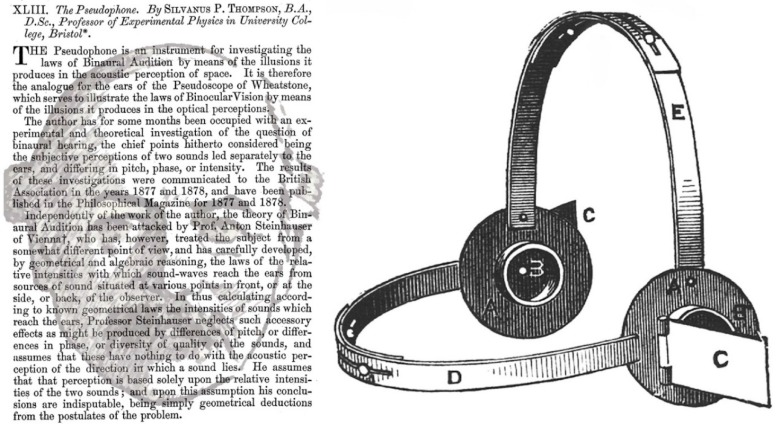
(**Left**), *Pseudophonist* by Nicholas Wade. Silvanus Phillips Thompson is shown in the opening page of his article describing the pseudophone [36]; (**Right**), Thompson’s illustration of the instrument which: “consists of a pair of ear-pieces, A A, furnished with adjustable metallic flaps or reflectors of sound, C C, which can be fitted to the ears by proper straps, D and E, and can be set at any desired angle with respect to the axis of the ears, and can also be turned upon a revolving collar about that axis so as to reflect sounds into the ears from any desired direction” ([36], p. 387).

Thompson (Figure 8) was able to examine binaural direction by manipulating the source of the stimulus by means of his pseudophone. As with other binaural devices its invention was modelled on similar binocular instruments, in this case Wheatstone’s pseudoscope which reversed the disparities in each eye by means of prisms [29]. Rather more elaborate versions of pseudophones were devised before electronic control of binaural stimuli was introduced.

**Figure 9:**
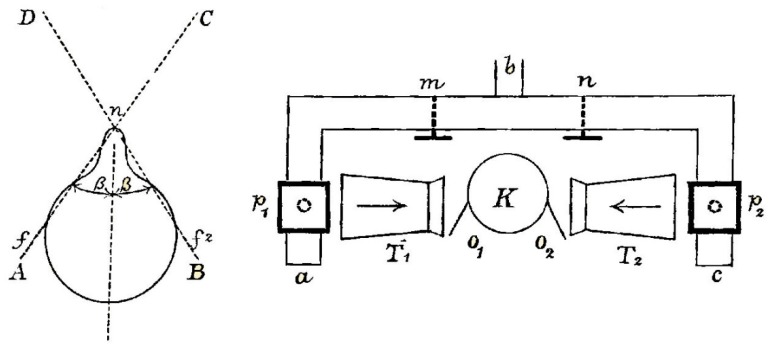
Diagrams from Steinhauser [38] showing (**left**) the head from above and the limits of direct and mixed binaural audition and (**right**) his homophone model which presented sounds from organ pipes to each ear (*O*_1_ and *O*_2_).

Steinhauser built his theory of binaural hearing on an analysis of auditory localization: “*the direction in which a source of sound is situated may be estimated by the different intensities with which a sound is perceived in the two ears*” ([38], p. 186). The pinna of each ear played a significant role in the differential intensities reaching the auditory canal, as he indicated graphically (Figure 9), and determined trigonometrically. Sounds within the angle D*n*C were referred to as direct because they were projected to each ear whereas those within the angles A*n*D or B*n*D were called mixed due to the direct stimulation of one ear relative to the other; indirect stimulation was from behind the head. He divided the whole of auditory space into three regions: “*in front*, the region of direct hearing; at *the two sides*, the regions of mixed hearing; and *at the back*, the region of indirect hearing” ([39], p. 272). In addition, he illustrated a model of the homophone “which is for the ears the analogue of the stereoscope for the eyes” (Steinhauser, 1879a, p. 188) but he only applied it for a theoretical analysis of binaural direction.

**Figure 10:**
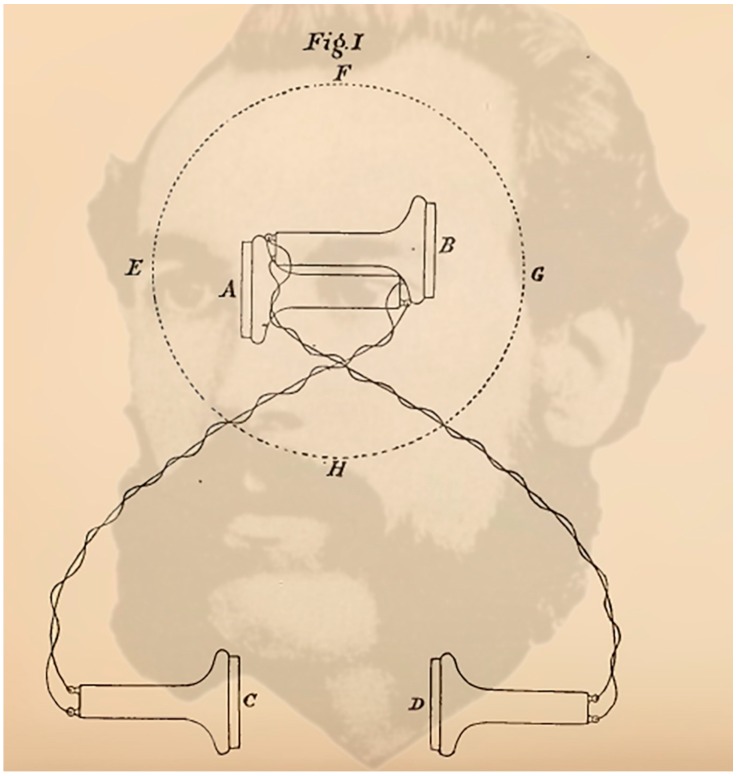
*Telephonist* by Nicholas Wade. A portrait of Alexander Graham Bell combined with the arrangement of telephones in the studies described by Bell [57].

Alexander Graham Bell (Figure 10) also performed an experiment similar to that of Venturi but with the added technical sophistication of the telephone. He was aware that “the difference between monaural and binaural audition is especially well marked when we attempt to decide by ear the locality of a particular sound” ([57], p. 169). In order to pursue this difference experimentally he set up an arrangement of telephones receiving signals from one room and listened to in another. Telephone A was connected to C and B to D. They were separated by about the distance between the ears. A and B were in one room (EFGH) while C and D were in another. Speech from a person moving around room EFGH could be heard by the listener using either C and D or C or D alone. The listener was required to indicate the location within the room of the speaker. He concluded that “the direction of a source of sound is less perfect by a single ear than by both ears” ([57], p. 175). He also found, as with Venturi and Lord Rayleigh, that binaural sounds could be localized in the auditory axis but that those from straight ahead or behind were confused.

Bell’s interests in binaural audition were influenced by Thompson’s experiments on binaural beats. Thompson had used telephones in some of his lectures and corresponded with Bell about them [58]. It was during a visit to London that Bell started to examine the phenomena of stereophonic hearing in a manner similar to that applied to stereoscopic vision: “There seems to be a one-sidedness about sounds received through a single ear, as there is about objects perceived by one eye. When both ears are employed simultaneously, a sort of stereoscopic effect of audition is perceived. Sounds assume a “solidity” (if I may use the expression) which was not perceptible so long as one ear alone was employed. The difference between monaural and binaural audition is especially well marked when we attempt to decide by ear the locality of a particular sound” ([57], p. 169). Bell went on to describe “that the stereophonic phenomena of binaural audition might be produced artificially by the telephone, in like manner as the peculiarities of binocular vision are produced by the stereoscope” (pp. 169–170). His experiments with paired independent telephone signals supported the superiority of binaural over monaural localization. Bell was also intrigued by Thompson’s pseudophone about which they also corresponded.

Pierce [15] made two speculations regarding the disparity between studies of visual and auditory space. The first was: “*that the ear possesses no spread-out surface such that the arrangement of stimulations upon it may represent spatial relations of the external world*. The ear, that is, possesses nothing comparable to the retina, the articular surfaces or the skin” (p. 16, original italics). A second distinction could have been the extent of movement of the sense organs themselves: “A second reason why an auditory space has been denied would seem to be *that the ear is unprovided with a muscular apparatus of such a complexity as to enable it to focus itself*, as it were, *upon sounding bodies lying in various directions from it*. That is, there is no possibility of having a widely varying set of movements of the auditory organ which may correspond to the various positions of this organ necessary for most distinct hearing” (p. 18, original italics).

Further study was inhibited by debates regarding absence of spatiality in hearing. When auditory localization was examined at the end of the 19th century it was dominated by controversies over whether intensity or temporal differences serve as cues, but there were researchers also concerned with non-theoretical experimental questions. Rayleigh [40] proposed a duplex theory of binaural localization: it was possible due to inter-aural differences in intensity and time of arrival of the sounds. Later it was recognized that the two bases for localization operated at different frequency bands; one for high frequency tone serving as an intensity cue and the other for low frequency tones serving as a temporal cue. There now exists a large body of binaural phenomena but they are based on relatively recent studies [17].

## 4. Conclusions

The study of vision has been dominated by cataloguing observations whereas hearing has focused on defining the stimulus—sound. The physical characteristics of sound were appreciated long before those of light. Thus, seeing and hearing were distinguished by knowledge of the sources of stimulation as well as by the concepts used to account for their reception. Spatial dimensions could be measured and manipulated in pictorial stimuli. In addition, it was appreciated that what could be seen with one eye differed slightly from that seen by the other. Hearing, on the other hand, is temporal and concepts of images were not incorporated into theories. Differences in the sounds experienced by one ear were rarely compared to those in the other. Fractionating time into smaller intervals proved much more difficult than fractionating space. Moreover, temporal resolution in hearing was much more acute than in seeing with the opposite applying to spatial resolution.

Visual direction has received attention since antiquity. After the appreciation of image formation in the eye, early in the 17th century, direction was defined in retinal terms. This raised problems for objects appearing in a single direction when using two eyes and led to the experiments by Wells in 1792 which led to the integration of eye position with binocular optical projections. Auditory direction received less observational, experimental, and theoretical attention until parallels between phenomena of binocular vision (like rivalry) were examined in the context of binaural hearing. In 1796 Venturi examined binaural direction as a consequence of his experiments on binocular color combination. Thereafter, instruments for investigating binaural direction were based on similar ones for binocular vision—such as the stereoscope/stethophone and pseudoscope/pseudophone.

## Figures and Tables

**Table 1:** Comparison of pages devoted to vision and hearing in books on the senses.

Book	Period Covered	Vision	Hearing
Theophrastus [1]	Early Greek	6	3
Beare [2]	Early Greek	81	37
Galen [3]	Roman	117	13
Kemp [4]	Medieval	10	1
Woolgar [5]	Late Medieval	43	21
Aquapendente [6]	16th century	133	38
Bell [7]	18th century	148	80
Schäfer [8]	19th century	122	56
Luciani [9]	Early-20th century	171	75
Boring [10]	Mid-20th century	214	124
Held, et al. [12]	Late-20th century	580	66
Barlow and Mollon [13]	Late-20th century	204	93
Kandel, et al. [14]	20th century	98	35
Goldstein [15]	Early-21st century	214	67
